# Expression of importin-α isoforms in human nasal mucosa: implication for adaptation of avian influenza A viruses to human host

**DOI:** 10.1186/s12985-016-0546-y

**Published:** 2016-06-04

**Authors:** Khwansiri Ninpan, Ornpreya Suptawiwat, Chompunuch Boonarkart, Peerayuht Phuangphung, Sakda Sathirareuangchai, Mongkol Uiprasertkul, Prasert Auewarakul

**Affiliations:** Department of Microbiology, Faculty of Medicine Siriraj Hospital, Mahidol University, Bangkok, 10700 Thailand; Department of Forensic Medicine, Faculty of Medicine Siriraj Hospital, Mahidol University, Bangkok, Thailand; Department of Pathology, Faculty of Medicine Siriraj Hospital, Mahidol University, Bangkok, Thailand

**Keywords:** Influenza A virus, Importin-α1 isoform, Importin-α3 isoform, Importin-α7 isoform, Human nasal mucosa, Human lung cells, Human respiratory tract, Interspecies barrier

## Abstract

**Background:**

Transportation into the host cell nucleus is crucial for replication and transcription of influenza virus. The classical nuclear import is regulated by specific cellular factor, importin-α. Seven isoforms of importin-α have been identified in human. The preference of importin-α3 of avian influenza virus and -α7 isoform of human strains during replication in human cells was previously identified. In addition, both avian and human influenza viruses were shown to use importin-α1 isoform for their replication.

**Finding:**

The mRNA levels of importin-α1, −α3, and –α7 isoforms in human respiratory tract was determined by real-time RT-PCR. The results indicate that mRNA level of importin-α7 was significantly higher than that of importin-α1 (*p*-value < 0.0001) and importin-α3 (*p*-value < 0.0001) isoforms in human nasal mucosa while importin-α1 was detected as the highest expression importin-α isoform in lung tissues.

**Conclusions:**

These results may explain the preference of importin-α7 isoforms in seasonal influenza viruses in human upper respiratory tract and may suggest a selective pressure toward importin-α7 in human respiratory tract infection of an avian virus.

## Findings

Influenza’s lifecycle requires nuclear import of the viral ribonucleoproteins (vRNPs) for viral replication and transcription, however, the large viral ribonucleoprotein (vRNPs) complexes exceed the size limitation of nuclear pore of the host cells [1, 2]. Therefore, translocation across host cell nuclear membrane depends on specific cellular factors of karyopherins superfamily, importin-α and -β. The classical nuclear import is regulated by importin-α as an adaptor protein that links the nuclear localization signal (NLSs) of imported molecules to importin-β which in turn mediates the transportation across nuclear envelope [3–5]. While the human genome encodes only a single importin-β gene, seven importin-α genes have been described for seven isoforms of importin-α: α1, α3, α4, α5, α6, α7 and α8 [6].

It has been previously shown that seasonal and avian influenza viruses require different importin-α isoforms for this process. Silencing of importin-α7 in human lung cells reduced seasonal influenza virus replication, while growth of avian strains was limited in importin-α3 silenced cells. On the other hand, growth of both avian and seasonal influenza viruses was reduced by importin-α1 silencing. Moreover, a reduction of viral load of human influenza viruses in lung was observed in importin-α7-knockout mice [7]. Taken together, importin-α proteins may act as another possible barrier during influenza viruses adaptation to the new host species.

The fact that mammalian influenza viruses showed the preference of importin-α7 isoform during viral replication prompted us to ask whether human upper airway expresses importin-α isoforms in resemblance to the known pattern of susceptibility of human respiratory tract tissues to human influenza viruses. It was previously shown that importin-α1 and -α7 was widely expressed in most human tissues [8], while importin-α3 was expressed in lung, testis, ovary, small intestine, heart, skeletal muscle, and pancreas but less detectable in kidney, thymus, colon and peripheral blood leukocytes [9]. Although distributions of importin-α isoforms were presented in several studies, the difference between expression of importin-α1, importin-α3 and –α7 in human upper airway tissue has not yet been definitely answered.

In this study, the mRNA copy numbers of importin-α1, −α3 and –α7 isoforms in human respiratory tract were determined. The nasal mucosae and lung tissues were derived from 10 and 5 autopsy cases, respectively, who died accidentally at the age of 20–60 years old. This part of the study was approved by the Ethics Committee of Siriraj Institutional Review Board, Faculty of Medicine Siriraj Hospital, Mahidol University (Protocol number 805/2554 (EC2)). The parents, relatives or spouse of the dead person was informed by participant information sheet and signed in the consent forms for participation voluntarily.

**Fig. 1:**
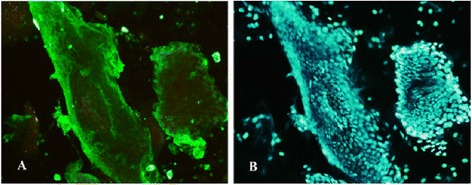
Immunofluorescence staining of nasal mucosae samples (20×). Double immunofluorescence staining was performed by using anti-human cytokeratin antibody (**a**) to detect the proportion of epithelial cells and Hoechst (**b**) for nuclear detection

In order to define the expression level of importin-α1, −α3 and –α7 isoforms in human nasal mucosa, epithelial cells of nasal mucosae were collected by blade scraping. Then, the proportion of epithelial cells in mucosal samples was defined by immunofluorescence assay using anti-human cytokeratin antibody. Approximately 85 % of collected cells were shown to be epithelial cells by positive cytokeratin staining in all mucosal samples as indicated in Fig. 1.

The amounts of importin-α1, −α3 and –α7 mRNA copy number in human nasal mucosa were obtained by extrapolation of the cycle number against the standard curve. Importin-α1,-α3 and –α7 mRNA expression levels were normalized with glyceraldehyde 3-phosphate dehydrogenase (GAPDH) expression. The correlation coefficients of the standard curves were 0.992 for GAPDH, 0.9941 for importin-α1, 0.951 for importin-α3, and 0.94 for importin-α7 mRNA expression (data not shown). The RT-PCR was performed in three independent experiments with a total of 5 repeats, excepted for the detection of importin-α1 mRNA expression level in nasal mucosae because of limited availability of the sample. Importin-α1 measurement was done in duplicate of 2 nasal mucosa samples while the others 3 samples were done in 5 repeats as in other experiments. Statistical analysis was performed using unpaired t-test. A *p*-value of <0.05 was considered statistically significant.

In order to ensure the RNA quality of individual samples, 1 μg of each extracted RNA were amplified with GAPDH specific primer. The mRNA expression levels of GAPDH were comparable among sample. The mean and standard deviation (SD) of copies numbers were found to be 5.35 × 10^2^ copies with SD of 1.23 × 10^1^ copies in all nasal mucosa samples.

**Fig. 2:**
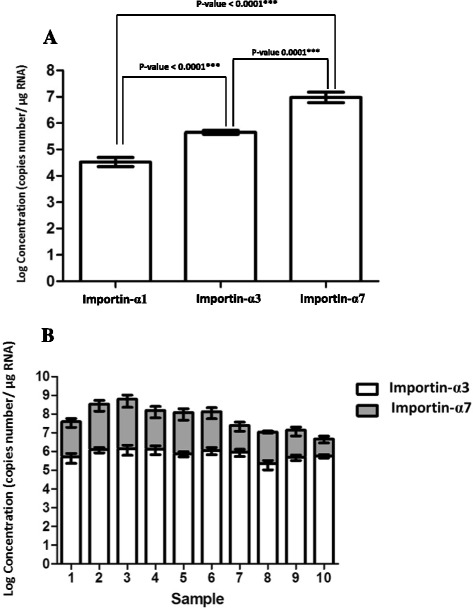
**a** Average mRNA expression level of importin-α1 (*n* = 5), −α3 (*n* = 10) and importin-α7 isoform (*n* = 10) in human nasal mucosae. **b** Individual mRNA expression ratios between importin-α3 and importin-α7 isoform. The bars represent geometric mean ± SEM. Statistical analysis was performed using unpaired t-test. A *p*-value of <0.05 was considered statistically significant

Figure 2a indicates the expression level of importin-α1, −α3 and -α7 mRNA from 10 individuals. The result showed that importin-α7 had a significant higher mRNA level in human nasal mucosa than importin-α1 and -α3 isoform with approximately 10^7^ copies/μg RNA, ten times greater than that of importin-α3 isoforms (*p*-value < 0.0001), and one hundred times greater than the expression level of importin-α1 (*p*-value < 0.0001). The expression ratio between importin-α7 and importin-α3 isoform of each samples were shown in Fig. 2b. This higher expression of importin-α7 isoform and the scarcity of importin-α1 and –α3 in nasal mucosa may provide a selective pressure for importin-α7 preference in human influenza viruses.

**Fig. 3:**
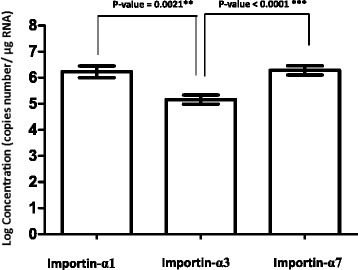
The mRNA expression level of importin-α1, −α3 and importin-α7 isoform in human lung tissues from 5 individuals. The RT-PCR was performed in three independent experiments with a total of five repeats. The bars represent geometric mean ± SEM. Statistical analysis was performed using unpaired t-test. A *p*-value of <0.05 was considered statistically significant

Besides human upper respiratory tract tissue, nasal mucosa, the expression level of importin-α1, −α3 and -α7 isoforms were also tested in human lower respiratory tract, lung tissues. The expression level of importin-α isoforms was determined in grinded lung tissues from 5 individuals. While the expression level of importin –α3 and –α7 isoforms were comparable to that in nasal mucosa, approximately one hundred times higher expression of importin-α1 was found in lung tissues when compared with nasal mucosa (Fig. 3).

This study provides an explanation for selective pressure driving the previously described human influenza virus preference for importin-α7, the interspecies barrier caused by importin alpha preference, and the different tissue tropism between seasonal and avian influenza viruses. Higher expression level of importin-α7 isoforms in human nasal mucosa than that of importin-α1 and -α3 isoforms with the known tissue distribution of the α2,3- and α2,6-linked sialic acid as the viral entry receptor may indicates a two-fold interspecies barrier at the cell and nuclear entry steps with similar tissue distribution.
